# 43 kDa Glycoprotein (gp43) from *Paracoccidioides brasiliensis* Induced IL-17A and PGE2 Production by Human Polymorphonuclear Neutrophils: Involvement of TLR2 and TLR4

**DOI:** 10.1155/2019/1790908

**Published:** 2019-11-26

**Authors:** Taiane Priscila Gardizani, Amanda Manoel Della Coletta, Graziela Gorete Romagnoli, Rosana Puccia, Ana Paula Moreira Serezani, Ângela Maria Victoriano de Campos Soares, Luciane Alarcão Dias-Melicio

**Affiliations:** ^1^Laboratory of Immunopathology and Infectious Agents (LIAI), Experimental Research Unit (UNIPEX), Medical School of Botucatu (FMB), São Paulo State University (UNESP), Av. Professor Montenegro, s/n, Distrito de Rubião Júnior, 18618-687 Botucatu, São Paulo, Brazil; ^2^Department of Medicine, University Center of Adamantina, Av. Francisco Bellusci, 1000, Distrito Industrial Otavio Gacazzi, 17800-000 Adamantina, São Paulo, Brazil; ^3^Department of Microbiology and Immunology, Institute of Biosciences (IB), São Paulo State University (UNESP), Av. Professor Montenegro, s/n, Distrito de Rubião Júnior, 18618-687 Botucatu, São Paulo, Brazil; ^4^Departamento de Microbiologia, Imunologia e Parasitologia, Escola Paulista de Medicina-Universidade Federal de São Paulo, Rua Botucatu, 862, 04023062 São Paulo, São Paulo, Brazil; ^5^Division of Allergy, Pulmonary, and Critical Care Medicine, Department of Medicine, Vanderbilt University Medical Center, Nashville, TN, USA; ^6^Department of Pathology, Medical School of Botucatu (FMB), São Paulo State University (UNESP), Av. Professor Montenegro, s/n, Distrito de Rubião Júnior, 18618-687 Botucatu, São Paulo, Brazil

## Abstract

The glycoprotein gp43 is the major antigenic/diagnostic component of *Paracoccidioides brasiliensis*, one of the etiologic agents of paracoccidioidomycosis (PCM). Gp43 has protective roles in mice, but due to adhesive properties, this glycoprotein has also been associated with immune evasion mechanisms. The present study evaluated gp43 interaction in vitro with Toll-like receptors 2 and 4 (TLR2 and TLR4) present in polymorphonuclear neutrophils (PMNs) from healthy human individuals and the consequent modulation of the immune response through the expression and release of cytokines and eicosanoids. PMNs were incubated in the absence or presence of monoclonal antibodies anti-TLR2 and anti-TLR4 (individually or in combination) before gp43 stimulation. Then, PMNs were analyzed for the expression of both surface receptors and the detection of intracytoplasmic IL-17A and IL-4 using flow cytometry, while the production of PGE2, LTB4, IL-6, IL-10, IL-12, IFN-*γ*, and TNF-*α* was evaluated in the supernatants by enzyme-linked immunosorbent assay (ELISA). Our results showed that gp43 increased TLR2 and TLR4 expression by PMNs and induced PGE2 and IL-17A via TLR4 and TLR2, respectively. Thus, our data suggest that gp43 from *P. brasiliensis* might modulate host susceptibility to the fungal infection by affecting PGE2 and IL-17A production.

## 1. Introduction

Paracoccidioidomycosis (PCM) is a systemic granulomatous disease prevalent in Latin America. The etiological agents belong to the *Paracoccidioides* gender of thermodimorphic fungi, among which *Paracoccidioides brasiliensis* is the most studied species [1–5]. PCM infection initiates through the inhalation of environmental mycelium propagules that reach the pulmonary alveoli, where they can only survive upon transformation into yeasts, a process mediated by the body's temperature [6, 7]. Disease establishment, progression, and severity depend both on fungi virulence factors as well as the host's immunological response.

*P. brasiliensis* exhibits in the cytoplasm, and along the cell wall, a 43-kDa glycoprotein (gp43) that is considered the main fungal antigen. It is secreted by the fungus and frequently found in the serum of PCM patients [8–10]. Due to its adhesive properties, gp43 is associated with the fungal virulence factors, induction of cell apoptosis, and modulation of the local and systemic inflammatory response, which may contribute to fungal infection and dissemination to other tissues [11–17]. Inhibition of gp43 expression in genetically modified *P. brasiliensis* resulted in a less severe infection in experimentally infected mice due to diminishing adherence of the fungi to host cell proteins, increased yeast cell phagocytosis, and consequent production and action of proteases responsible for inhibiting fungal tissue diffusion [18]. The first cell types recruited to the infection sites are polymorphonuclear neutrophils (PMNs), which remain in the lesion to form a suppurative granuloma in the chronic phase of this mycosis [19, 20]. Effector and modulatory mechanisms of PMNs are dictated by the recognition of conserved structures presented by the microorganism denominated pathogen-associated molecular patterns (PAMPs), which are recognized by pattern-recognition receptors (PRRs) [21, 22]. Among the PRRs involved in fungal recognition, Toll-like receptors (TLRs) are a family of single-pass type I transmembrane-spanning proteins [23]. Following TLR binding to PAMPS, an intracellular signaling pathway is triggered, promoting the release of inflammatory mediators and modulation of innate and adaptive immunity [24–30]. PMNs can recognize *P. brasiliensis via* both TLR2 and TLR4, leading to the production of TNF-*α*, IL-8, IL-12, IL-10, prostaglandin E2 (PGE2), and leukotriene (LTB4) [24, 26, 27, 29–31]. Although most studies have evaluated PMN interactions with yeast or the mycelium of *P. brasiliensis*, the interactions and effects of gp43 in PMNs remain unknown. Therefore, the present study assessed whether gp43 is recognized by TLR2 and TLR4 on the surface of human PMNs, consequently modulating the production of immunomodulatory cytokines and eicosanoids.

## 2. Materials and Methods

### 2.1. Subjects

Heparinized venous blood was obtained from 7 voluntary healthy women, with age ranging from 19 to 37 years-old, after signing informed consent forms. This study was approved by the Research Ethics Committee from Botucatu Medical School, São Paulo State University (UNESP) (CAAE number: 80098817.8.0000.5411).

### 2.2. Isolation of Peripheral Blood Polymorphonuclear Neutrophils

Forty microliters of blood were layered above density gradients such as Histopaque 1119 g/ml, followed by Histopaque 1083 g/ml (Sigma-Aldrich, St. Louis, MO, USA), and centrifuged at 405 g, for 30 min, at room temperature. The interface layers of polymorphonuclear neutrophils (PMNs) were collected; erythrocytes were lysed with 0.1% NaCl, and cell viability was assessed with trypan blue dye (95% viability). PMNs were then suspended in RPMI 1640 medium supplemented with 5% heat-inactivated fetal bovine serum and 1% gentamicin (Cultilab, Campinas, São Paulo, Brazil; Sigma-Aldrich, St. Louis, MO, USA; and Schering-Plough, São Paulo, São Paulo, Brazil, respectively) and adjusted to 2 × 10^6^ cells/ml in 96-well plates or 1 × 10^6^ cells/ml in 48-well plates. Microcultures were intended to evaluate the expression of the receptors and the intracytoplasmic cytokines, whereas macrocultures were used in the cytokine and eicosanoid assays. All assays were performed in duplicate.

### 2.3. Purification of gp43

Gp43 was purified from *P. brasiliensis* (Pb339 strain) culture supernatants by affinity chromatography in Affi-Gel 10 columns (Bio-Rad) containing the anti-gp43 monoclonal antibody MAb17c [9], which recognizes all gp43 isoforms [32], as previously described [33].

### 2.4. TLR2 and TLR4 Expression and Blockage of the Receptor

The experimental protocol was conducted as described by Nakaira-Takahagi et al., with some adaptations [34]. PMNs were initially treated or not with monoclonal antibodies anti-human TLR2 (0.8 *μ*g/10^5^ cells) (BioLegend, San Diego, CA, USA) and mouse anti-human TLR4 (2 *μ*g/10^5^ cells) (BD Pharmingen™, Franklin Lakes, NJ, USA), individually or in combination, for 2 h in a 5% CO_2_ atmosphere at 37°C. After the receptor blockade, cells were incubated in the presence of gp43 (20 ng/ml) or in its absence (control culture) for 4 hours at the same conditions described above. Supernatants were collected, centrifuged, and stored at -20°C for measuring IL-6, IL-10, IL-12, IFN-*γ*, PGE2, and LTB4 levels. PMNs were then incubated with PerCP anti-human CD16 antibody, FITC anti-human CD282 (TLR2) antibody (both antibodies from BioLegend, San Diego, CA, USA), and PE mouse anti-human TLR4 antibody (BD Pharmingen™, Franklin Lakes, NJ, USA) for 20 minutes at 4°C in the dark. Receptor expression was determined by FACSCanto II flow cytometry (BD, San Diego, CA, USA) using the FACSDiva software. The standard acquisition was set to 25,000 events, and cells were gated based on size (forward scattered (FSC)), granularity (side scattered (SSC)), and fluorescence parameters (PerCP-CD16+ and/or FITC-TLR2+ and/or PE-TLR4+). Data were analyzed with FLOWJo software.

### 2.5. Intracytoplasmic Detection of IL-4 and IL-17A by Flow Cytometry

After the receptor blockade, PMNs were incubated with gp43 (20 ng/ml) or LPS (1 *μ*g/ml) as a positive control (data not shown) and, at the same time, with brefeldin A (5 mg/ml, BioLegend, San Diego, CA), for 4 h, to perform intracytoplasmic cytokine detection. After that, cells were incubated with PerCP anti-human CD16 antibody for 20 min at 4°C. Then, cells were centrifuged, the supernatant was carefully removed, and cell pellets were resuspended in 100 *μ*l of reagent A from the FIX&PERM kit (Nordic MUbio, Susteren, The Netherlands) and incubated at 4°C for 15 min. The suspension was centrifuged, supernatants were removed, and cells were incubated with 100 *μ*l of reagent B from the FIX&PERM kit in the presence of PE mouse anti-human IL-4 or PE mouse anti-human IL-17A antibody (BD Pharmingen™, Franklin Lakes, NJ), individually, for 30 min at 4°C. Finally, cells were centrifuged to remove the supernatant and resuspended in 100 *μ*l of fixing solution. All steps were performed in the dark to protect the cells from light exposure. The intracytoplasmic expression of IL-4 and IL-17A was determined by FACSCanto II flow cytometry, using FACSDiva software. The standard acquisition was set to 30,000 events, and cells were gated based on size (forward scattered (FSC)), granularity (side scattered (SSC)), and fluorescence parameters (PerCP-CD16+ and PE-IL-4+ or PE-IL-17A+). Data were analyzed with FLOWJo software.

### 2.6. Determination of Cytokines and Eicosanoids in Cultures Supernatant

Concentrations of IL-6, IL-10, IL-12, IFN-*γ*, and TNF-*α* were detected using Human IL-6, IL-10, and IL-12 TNF-*α* ELISA Sets (BD OptEIA™, BD, San Diego, CA, USA) and IFN-*γ* by DuoSet ELISA (R&D Systems, Minneapolis, MN, USA). PGE2 and LTB4 levels were measured using a competitive ELISA kit from Cayman Chemical Company (Ann Arbor, MI, USA). All assay types were conducted according to the manufacturer's protocol.

### 2.7. Statistical Analysis

Results were analyzed using GraphPad Prism (GraphPad Software Inc., San Diego, CA, USA), and the significance level was set at *p* < 0.05. Nonparametric data were presented as median and analyzed using the Friedman test, followed by the posttest of the multiple Dunn comparisons. Parametric data were expressed as mean ± SD and analyzed by Analysis of One-Way Variance (ANOVA) and Tukey's multiple comparison posttest. The Shapiro-Wilk was the normality test used.

## 3. Results

### 3.1. Expression of TLR2 and TLR4 following PMN Stimulation with gp43

**Figure 1:**
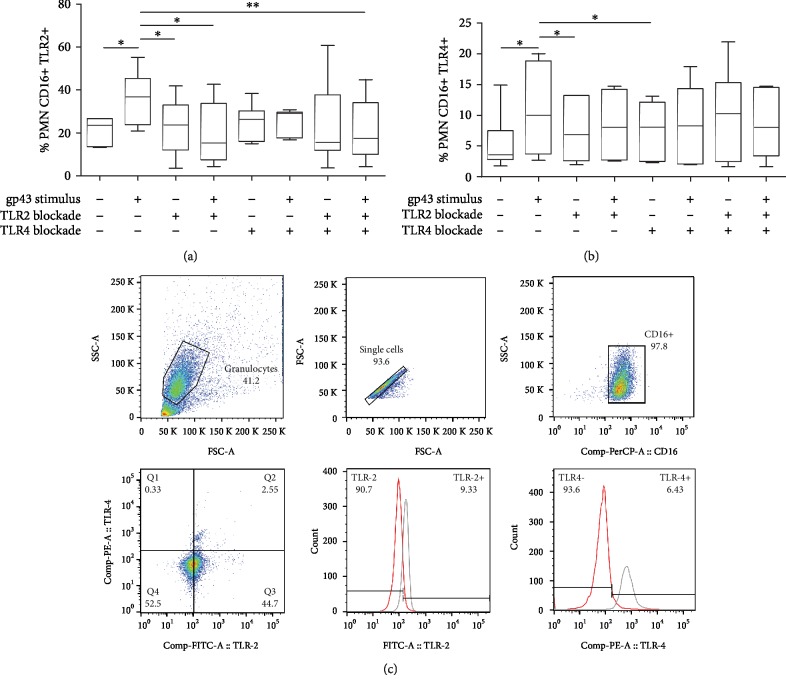
Percentage of TLR2 (a) and TLR4 (b) expression on the surface of CD16+ human polymorphonuclear neutrophils (PMNs). PMNs were incubated in the absence (control) or presence of gp43 (20 ng/ml) for 4 h, and PMNs were analyzed using flow cytometry. Results are presented as percentage of PMNs expressing TLR2 and TLR4 using box-and-whiskers (min to max) graphs with data from 7 women tested, considering *p* < 0.05. Statistically significant differences between groups are indicated as follows: ^∗^*p* < 0.05 and ^∗∗^*p* < 0.01. (c) Representative experiment showing the expression of PMN (control culture) CD16 PerCP+, TLR2 FITC+, and TLR4 PE+, and histograms display the percentage of TLR2 and TLR4 blockade (red line) and the remaining positive receptor expression (gray lines).

To test the hypothesis that gp43 interacts with either TLR2 or TLR4 from PMNs, we initially incubated human PMN cells with 20 ng of gp43 for 4 hours and analyzed the expression of these two receptors by flow cytometry.  Figure 1 shows a significant increase in TLR2+ (Figure 1(a)) and TLR4+ (Figure 1(b)) cells after stimulation with gp43 alone. We then blocked TLR2 or TLR4 using a monoclonal antibody (anti-TLR2 or anti-TLR4) prior gp43 stimulation, and we observed that the percentage of TLR2+ and TLR4+ cells remained similar to nonstimulated control cells (Figure 1(a) and 1(b)). TLR2 and TLR4 expression also remained identical to the control group when both receptors were blocked simultaneously and then incubated with glycoprotein (Figure 1(a)). These data indicate that gp43 is sufficient to induce TLR2 and TLR4 expression by human PMN cells.

### 3.2. Role of TLR2 and TLR4 in PGE2 and LTB4 Production by PMNs Incubated with gp43

**Figure 2:**
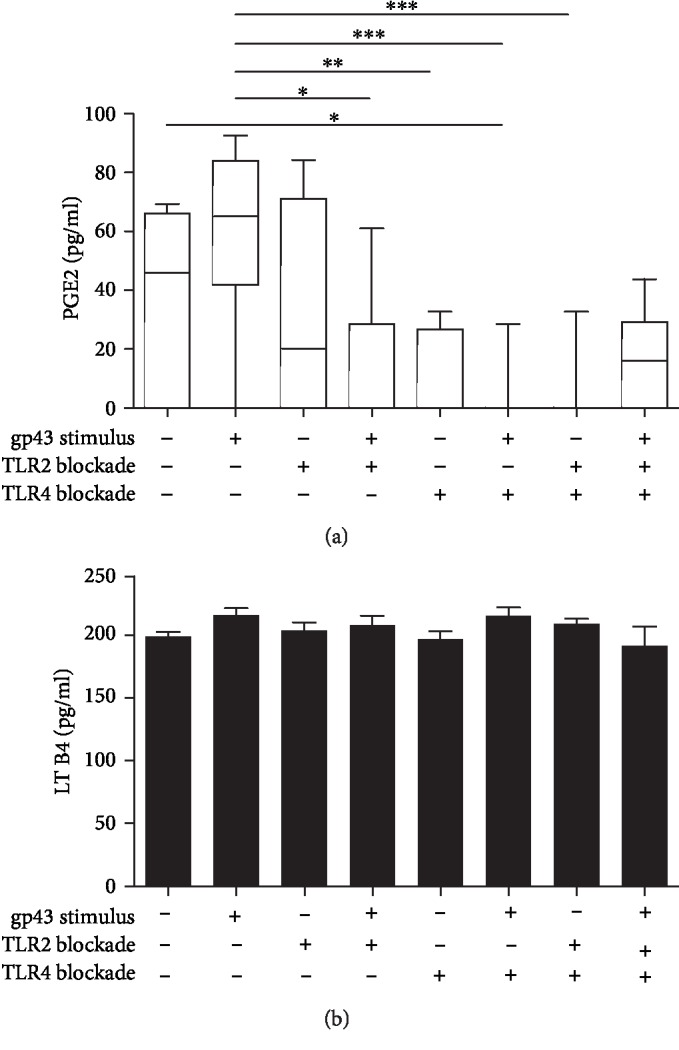
Involvement of TLR2 and TLR4 in PGE2 (a) and LTB4 (b) production by human polymorphonuclear neutrophils (PMNs) stimulated with gp43 from *P. brasiliensis*. Cells were incubated in the absence or presence of anti-TLR2 and anti-TLR4 antibodies, individually or in combination for 2 h. PMNs were then stimulated or not with gp43 (20 ng/ml) during 4 h, and eicosanoid concentrations (pg/ml) were measured by competitive ELISA in the culture supernatants. Results are expressed using a box-and-whiskers (min to max) graph with data from 7 women tested, considering *p* < 0.05 (a) or mean ± SD (b), considering *p* < 0.05. Statistically significant differences between groups are indicated as follows: ^∗^*p* < 0.05, ^∗∗^*p* < 0.01, and ^∗∗∗^*p* < 0.001.

To investigate whether TLR2 or TLR4 are involved in the production of PGE2 by PMNs during the recognition of gp43, we then measured PGE2 in the supernatant of cells stimulated or not with gp43 and incubated or not with anti-TLR2 and anti-TLR4 monoclonal antibodies. Our results showed that TLR4 is the main receptor involved in the production of PGE2 by PMNs. PMNs incubated with gp43 showed a slightly increased production of PGE2 than unstimulated cells. Blocking TLR2 alone or simultaneously TLR2 and TLR4 resulted in a low production of PGE2 by gp43-stimulated PMNs when compared to control unstimulated cells (Figure 2(a)). However, after TLR4 blockage, PGE2 levels substantially declined in the presence of gp43.

To determine whether other eicosanoids besides PGE2 are induced during gp43 recognition, we evaluated the levels of leukotriene B4 (LTB4) in the supernatant of PMN-stimulated cells. However, gp43 did not influence LTB4 production by PMNs, and contrary to PGE2, no difference was observed in LTB4 levels after TLR2 and TLR4 blockage (Figure 2(b)).

### 3.3. Role of TLR2 and TLR4 in IL-17A and IL-4 Intracytoplasmic Detection in PMNs Stimulated with gp43

**Figure 3:**
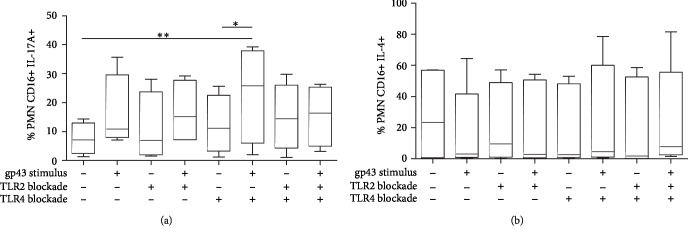
Percentage of human polymorphonuclear neutrophils (PMNs) producing intracytoplasmic IL-17A (a) and IL-4 (b) after stimulation or not with gp43 from *P. brasiliensis* and the involvement of TLR2 and TLR4. Cells were incubated in the absence or presence of anti-TLR2 and anti-TLR4 antibodies, individually or in combination, for 2 h. PMNs were then stimulated or not with gp43 (20 ng/ml) during 4 h and intracellular IL-4 and IL-17 were analyzed by flow cytometry. Results are presented as the percentage of PMNs expressing IL-17A (a) and IL-4 (b) using box-and-whiskers (min to max) graphs with data from 7 women tested, considering *p* < 0.05. (a) Statistically significant differences between groups are indicated as follows: ^∗^*p* < 0.05 and ^∗∗^*p* < 0.01.

Since IL-17A has an important role in protecting against fungal infections and given that PMNs are capable of producing IL-17 during infections, we investigated whether this cytokine is produced following gp43 stimulation. Thus, IL-17A was measured intracellularly in PMNs (CD16+ cells) coincubated or not with gp43, and our data demonstrated that IL-17A production involved the participation of TLR2 (Figure 3(a)). Gp43 alone resulted in a slight increase in the percentage of IL-17-producing cells when compared to nonstimulated PMNs. Moreover, blockage of TLR2 or TLR4 alone did not alter the percentage of IL-17+ cells (Figure 3(a)). However, PMNs cultured with anti-TLR4 and stimulated with gp43 showed an increased percentage of IL-17-producing PMNs when compared with unstimulated control cells, suggesting the participation of TLR2 in the production of IL-17A by gp43-stimulated neutrophils. To confirm this hypothesis, we blocked both TLR4 and TLR2 before gp43 stimulation and we observed that the percentage of IL-17A+ cells was similar to control unstimulated cells, confirming that TLR2 and TLR4 might compete for gp43 binding, and by blocking TLR4 responses, we can favor TLR2 actions in IL-17A production.

We next measured the capacity of PMNs to produce IL-4 following gp43 stimulation, a cytokine involved with high susceptibility to PCM infection. However, we did not observe differences in IL-4-producing PMNs independently of whether the cells were stimulated with gp43 or whether TLR2 or TLR4 were blocked during the culture conditions (Figure 3(b)).

### 3.4. Role of TLR2 and TLR4 in IL-6, IL-10, IL-12, IFN-*γ*, and TNF-*α* Production by PMNs Incubated with gp43

**Figure 4:**
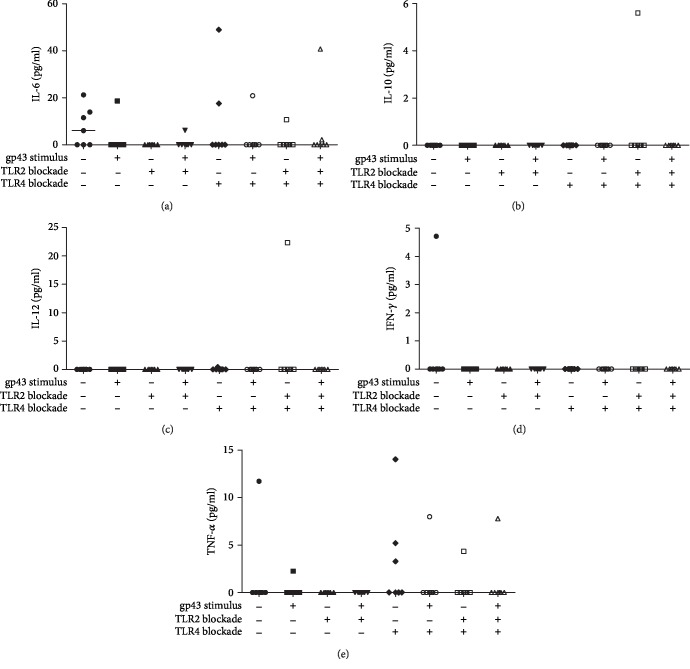
Involvement of TLR2 and TLR4 in the production of IL-6 (a), IL-10 (b), IL-12 (c), IFN-*γ* (d), and TNF-*α* (e) by human polymorphonuclear neutrophils (PMN) stimulated or not with gp43 from *P. brasiliensis*. Cells were incubated in the absence or presence of anti-TLR2 and anti-TLR4 antibodies, individually or in combination, for 2 h. PMNs were then stimulated or not with gp43 (20 ng/ml) during 4 h, and cytokine concentrations (pg/ml) were measured in the supernatant culture. Results are expressed as median using data from 7 women tested, considering *p* < 0.05.

Since the production of inflammatory cytokines frequently accompanies PCM infection, we next evaluated the effects of gp43 on the release of IL-6, IL-10, IL-12, IFN-*γ*, and TNF-*α* by human PMNs. Our results demonstrated that gp43 did not induce any of the cytokines evaluated (IL-6 (Figure 4(a)), IL-10 (Figure 4(b)), IL-12 (Figure 4(c)), IFN-*γ* (Figure 4(d)), and TNF-*α* (Figure 4(e))) after 4 hours of incubation with this glycoprotein. The data regarding IL-6 and TNF-*α* productions showed considerable variability between individuals, while IL-10, IL-12, and IFN-*γ* productions were not detected regardless of the presence or absence of gp43 and receptor blockage (Figure 4).

## 4. Discussion

Studies evaluating the role of PMNs in PCM have increased in the last few years due to the high number of PMNs in the initial phase of this fungal infection. To date, the studies have focused on modulatory functions of PMNs using whole yeast or the mycelium of *P. brasiliensis*. Here, we assessed the interaction of PMNs with gp43, the main immunodominant antigen of *P. brasiliensis*. In this context, we evaluated gp43 ability to modulate the expression of TLR2 and TLR4 by PMNs obtained from healthy donors and the subsequent release of inflammatory cytokines and lipid mediators.

Our data showed that PMNs exposed to gp43 upregulated both TLR2 and TLR4 expression. According to previous literature, the recognition of *P. brasiliensis* yeasts by PMNs involves different cell surface receptors, including dectin-1, mannose receptor (MR), and TLR2 or TLR4, which act collaboratively to provide mechanisms of resistance or susceptibility to fungal infection [24, 26, 27, 30]. Although we have not evaluated the involvement of other receptors besides TLRs, our data indicate that TLR2 and TLR4 might play essential roles in the modulation of PMN functions by gp43 during an active infection.

In this study, we also observed that PMNs produced both PGE2 and LTB4. However, while LTB4 was produced by gp43-stimulated or not stimulated PMNs, PGE2 appears to be induced in a TLR2- and TLR4-dependent manner. Balderramas et al. showed that PMNs release LTB4 when cells are incubated with *P. brasiliensis* yeasts, but neither TLR2 nor TLR4 were involved in LTB4 production [30]. Contrariwise, our data showed that gp43-induced PGE2 production required both TLR2 and TLR4, as evidenced by a substantial decrease in this eicosanoid production following blockage of these receptors. As seen in other studies, PGE2 production by PMNs in response to yeasts of *P. brasiliensis* involved not only TLR2 but also MR and dectin-1 receptors [26, 30]. Balderramas et al. [30] found spontaneous PGE2 levels similar to those that are present in this study, but increased levels of PGE2 were observed after challenge with two different fungal strains, a phenomenon mediated by simultaneous activation of different receptors. PGE2 production seemed to have a harmful role for hosts infected with *P. brasiliensis*. In murine PCM, PGE2 had an immunosuppressive effect at early steps of infection, by a mechanism dependent on IL-10 and IL-4 [35].

Moreover, PGE2 inhibition using indomethacin resulted in the production of hydrogen peroxide and TNF-*α* by monocytes and improved fungicidal activity [36, 37]. Additionally, studies showed that *P. brasiliensis* yeasts synthesize prostaglandins, being this lipid mediator required for fungal survival [38, 39]. Conversely, *P. brasiliensis* can affect dendritic cell maturation by inhibiting PGE2 production and causing inadequate production of IL-12p70 and TNF-*α* [40]. Thus, together, these findings suggest that *P. brasiliensis* might take advantage of PGE2 production for its survival as well as for the modulation of the immune response. We showed here that gp43 could be considered an essential component of *Paracoccidioides* responsible for the release of this lipid mediator mainly via TLR4. Per our findings, other studies have shown that *P. brasiliensis* utilizes TLR4 and TLR2 as a mechanism to infect human neutrophils and murine macrophages, taking advantage of the release of IL-10 and IL-8 following the activation of these receptors for increasing their multiplication inside these cells [25, 27]. Importantly, *P. brasiliensis* recognition via TLR4 in macrophages is associated with a more severe PCM in an experimental disease model [24].

We also observed a robust decrease in PGE2 production by gp43-activated PMNs after TLR4 blockage, indicating critical crosstalk between PGE2 and TLR4 during gp43 recognition. Other studies have shown the involvement of TLRs and PGE2 production. During gram-negative *Escherichia coli* infections, COX-2 and PGE2 are synthesized by vascular smooth muscle cells via TLR4 stimulation [41]. Also, it was identified that the stimulation of TLR7/8 by the synthetic agonist R-848 increases COX-2 expression in neutrophils [42]. However, a recent study showed that PGE2 restricts TLR4 signaling, indicating that more studies are warranted to determine the contribution of TLR4 to the control of PCM [43].

In this study, we also observed that gp43 results in IL-17A production by PMNs, most likely via TLR2, but not TLR4 activation. We showed that during gp43 stimulation, TLR4 blockage using a monoclonal antibody resulted in an increased percentage of IL-17A+ PMNs when compared to nonstimulated control cells. We then hypothesized that TLR2 and TLR4 compete for binding to gp43, and once the environment favors TLR2 binding to gp43, i.e., in the absence of TLR4 availability, the production of IL-17A is then significantly increased. Simultaneous blockade of TLR2 and TLR4 using monoclonal antibodies resulted in impaired augmentation of IL-17+ cells (different from blocking TLR4 alone), indicating a possible interaction between the two receptors in the modulation of IL-17A production by gp43. We have previously shown that monocytes from healthy individuals produced IL-17A after incubation with *P. brasiliensis* yeasts via activation of the Dectin-1 receptor [44]. However, until this moment, IL-17A production by PMNs incubated with yeasts or other components of *P. brasiliensis* had not been reported. Despite the controversial ability of neutrophils to produce this cytokine, our study detected intracellular IL-17A by PMNs stimulated or not with gp43. Thus, it is possible that TLR4 binding to gp43 drives both PGE2 production and inhibition of IL-17A production by PMNs, a mechanism that could lead to poor control of the fungal infection. However, the most severe form of PCM is characterized by a predominant Th17/Th22 response, along with the substantial participation of Th1 cells [45]. Thus, although high levels of IL-12 and IL-17 contribute to partial resistance to fungal infection, an exacerbated inflammatory response can be detrimental due to the induction of tissue damage and fibrosis [45–47].

Given that PMNs are observed in all stages of PCM lesions, the production of IL-17A by gp43-stimulated cells could intensify the inflammatory response and contribute to tissue damage. Loures et al. showed that TLR2 is a negative regulator of the pathogenic Th17 immunity, shifting the T cell responses to a balanced Th1/Th2 response modulated by Tregs [25]. In our study, gp43 stimulated PMNs to produce IL-17A only after blocking TLR4, suggesting that gp43 might take advantage of the TLR4/TLR2 interactions during IL-17 production to control inflammatory reactions during an active infection.

Although we have observed IL-17 production by gp43-stimulated human PMNs, we did not detect substantial levels of other inflammatory cytokines, including IL-4, IL-6, IL-10, IL-12, IFN-*γ*, and TNF-*α* following gp43 stimulation. Balderramas et al. reported the production of IL-12p40 and IL-10 after 4 hours of incubation with *P. brasiliensis* yeasts [30], indicating the participation of other fungal components in PMN activation. *P. brasiliensis* induces the production of all of these cytokines *in vitro* and *in vivo* modulating different arms of the immune system [27, 30, 31, 45]; but according to our data, gp43 seems not to participate in this process at least for a short period of incubation. This is a new and important study about PCM, demonstrating the recognition of gp43 by PMNs and its participation in PGE2 and IL-17A production, highlighting the use of the glycoprotein by *P. brasiliensis* to escape from host defense mechanisms.

## 5. Conclusion

Taken together, our data reinforce that gp43 from *P. brasiliensis* can function as an escape mechanism modulating the release of PGE2 and IL-17A by PMNs via TLR2/TLR4 interaction.

## Data Availability

All relevant data are within the paper and its supporting information files.
